# Bocavirus in Children with Respiratory Tract Infections

**DOI:** 10.3201/eid1709.110078

**Published:** 2011-09

**Authors:** Li Guo, Richard Gonzalez, Zhengde Xie, Hongli Zhou, Chunyan Liu, Chao Wu, Gláucia Paranhos-Baccalà, Guy Vernet, Kunling Shen, Qi Jin, Jianwei Wang

**Affiliations:** Author affiliations: State Key Laboratory for Molecular Virology and Genetic Engineering, Beijing, People’s Republic of China (L. Guo, Q. Jin, J. Wang);; Institute of Pathogen Biology, Beijing (L. Guo, R. Gonzalez, H. Zhou, C. Wu, Q. Jin, J. Wang);; Fondation Mérieux, Lyon, France (R. Gonzalez, G. Paranhos-Baccalà, G. Vernet);; Beijing Children’s Hospital, Beijing (Z. Xie, C. Liu, K. Shen)

**Keywords:** viruses, respiratory infections, detection, bocavirus, human bocavirus, respiratory tract infections, letter

**To the Editor:** Four species of human bocavirus (HBoV1–4) have been identified since 2005 (*1**–**4*). Several reports have documented that HBoV1 are prevalent in respiratory tract samples. Although there may be many asymptomatic carriers, HBoV1 has been shown to cause respiratory tract diseases (*1**,**5**,**6*). HBoV2 has mainly been detected in fecal samples and has been linked to gastroenteritis (*3**,**7**,**8*). HBoV3 and HBoV4 have recently also been detected in fecal samples (*3**,**4*), although no link to disease has been established for these 2 species.

In this study, we identified and characterized 3 HBoV species (HBoV1–3) detected in respiratory samples. Nasopharyngeal aspirates were collected from 1,238 children (784 boys and 454 girls) with acute lower respiratory tract infections hospitalized in Beijing Children’s Hospital from March 2008 through July 2010. Patients’ ages ranged from 1 month to 9 years (median 10.0 months, mean 32.1 months).

Viral nucleic acid was extracted from nasopharyngeal aspirates by using the NucliSens easyMAG system (bioMérieux, Marcy l’Etoile, France). We screened for HBoV1–4 by nested PCR with touch-down procedure using primers targeting the viral proteins (VP) 1/2 region (*4*). For HBoV-positive samples, we then quantified viral loads by real-time PCR (Technical Appendix). Additional viral infections were identified in all screened specimens as described (*9*). To avoid contamination, the PCR process (including master mixture preparation, nucleic acid extraction, reaction installation, and DNA amplifications) were performed in separate dedicated areas. Strict controls were used during the process of nucleic acid extraction and PCR analyses to monitor contamination. All PCR products were verified by sequence analysis.

We found 141 positive samples for HBoVs (11.4%) from patients ranging in age from 1 to 132 months (median 12.0 months, mean 19.8 months) (GenBank accession nos. HQ871520–HQ871650 and HQ871664–HQ871673). Among these samples, 131 (10.6%; patient ages 1–132 months, median 12 months, mean 19 months) were positive for HBoV1, 5 (0.4%; patients 1–113 months of age, median 7.2 months, mean 29.2 months) for HBoV2, and 5 (0.4%; 1–108 months of age, median 12.0 months, mean 30.4 months) for HBoV3 on the basis of sequence alignment and phylogenetic analysis of PCR amplicons. No specimens were positive for HBoV4. The number of samples positive for HBoV1, -2, and -3 in children <5 years old was 124/131 (94.7%), 4/5 (80%), and 4/5 (80%), respectively. Additional respiratory viruses were co-detected in 120/141 (85.1%) HBoV-positive patients (Table). An unanticipated finding was that real-time PCR only detected 1/5 positive sample for HBoV2 (viral load 4.87 × 10^9^ copies/mL) and 2/5 positive samples for HBoV3 (viral load 2.59 × 10^4^ and 4.1 × 10^2^ copies/mL). In contrast, we detected viral loads of 8.35 × 10^4^ to 1.28 × 10^9^ copies/mL in 5 randomly selected HBoV-1–positive samples.

**Table:** Distribution and clinical characteristics of HBoV species in 1,238 children with acute lower respiratory tract infections, Beijing, China, 2008–2010*

Parameters	HBoV1	HBoV2	HBoV3
No. (%) positive	131 (10.6)	5 (0.4)	5 (0.4)
Age range, mo	1–132	1–113	1–108
No. (%) patients <5 y of age	124 (94.7)	4 (80)	4 (80)
Age mean/median, mo	19/12	29.2/7.2	30.4/12
M/F	72/59	3/2	4/1
Clinical manifestations, no. (%)			
Fever	78 (59.5)	3 (60)	3 (60)
Cough	131 (100)	5 (100)	5 (100)
Sputum production	115 (87.8)	4 (80)	3 (60)
Wheezing	65 (49.6)	3 (60)	4 (80)
Runny nose	46 (35.1)	2 (40)	1 (20)
Diarrhea	9 (6.9)	0 (0)	1 (20)
Mean ± SD leukocyte count, ×10^9^/L	9.4 ± 5.3	7.56 ± 1.4	13.45 ± 6.1
Co-detection in samples, no. (%)			
Rhinovirus	58 (44.3)	1 (20)	2 (40)
Respiratory syncytial virus	42 (32.1)	2 (40)	2 (40)
Parainfluenza virus	24 (18.3)	0 (0)	1 (20)
Influenza virus	11 (8.4)	1 (20)	2 (40)
Conorovirus	15 (11.5)	0	0
Adenovirus	14 (10.7)	0	1 (20)
Human metapneumovirus	16 (12.2)	0	0
Enterovirus	2 (1.5)	1 (20)	0

*Detection rate for HBoV species, χ^2^ = 234.1, p <0.01. No HBoV4 was found. HBoV, human bocavirus.

The clinical diagnoses of patients providing HBoV-positive samples included pneumonia (63.1%), bronchitis (14.9%), bronchopneumonia (12.8%), and acute asthmatic bronchopneumonia (9.2%). No clinically significant differences were found between the signs and symptoms of patients with HBoV1, -2, and -3 (cough, sputum production, fever, runny nose, wheezing, and diarrhea). For patients positive for HBoV3, the major diagnoses were pneumonia (2/5), bronchopneumonia (2/5), and acute asthmatic bronchopneumonia (1/5). Main signs and symptoms included cough (5/5), wheezing (4/5), sputum production (3/5), fever (3/5), runny nose (1/5), and diarrhea (1/5). For patients positive for HBoV2, the diagnoses were pneumonia (4/5) and bronchopneumonia (1/5), and main signs and symptoms included cough (5/5), sputum production (4/5), wheezing (3/5), fever (3/5), and runny nose (2/5) (Table).

HBoV2 and HBoV3 were detected sporadically during the study. HBoV3 was detected in samples collected in July and October 2008, December 2009, and March and April 2010, whereas HBoV2 was detected in May, June, and October 2008, March 2009, and January 2010. The prevalence of HBoV1, which was responsible for 92.9% of the HBoV cases, was highest in January 2009 and April 2010.

Multiple-alignment analysis of sequences obtained in this study and reference sequences from GenBank (accession nos. FJ948861, NC_012564, EU918736, HM132056, and HQ152935) by using MEGA 4.0 (*10*) showed that amino acids in HBoV VP1/2 regions were 94.8%–100% identical among the strains of same species and 76.3%–100% identical among strains of different species. No obvious nucleotide and amino acid differences were found for the HBoV3 strains detected in respiratory samples and those in stool samples.

In summary, we report detection of genomic DNA of HBoV1, -2, and -3 in children with lower respiratory tract infections in China. The predominant HBoV species identified in our study was HBoV1. HBoV2 and HBoV3 appear to be present in much fewer positively identified cases and their viral load seems very low. For these latter viruses, however, low level mucosal contamination from the gastrointestinal tract cannot be entirely excluded in all cases. Further investigations are needed to confirm potential associations of HBoV2 and HBoV3 with acute lower respiratory tract infections, to determine their replication in the respiratory tract, and the viruses’ role in human disease.

## Supplementary Material

Technical Appendix Real-time PCR for Human Bocavirus.
