# Supplementary material for: Bocavirus in Children with Respiratory Tract Infections
Source: Emerg Infect Dis. 2011 Sep;17(9):1775–7. doi: 10.3201/eid1709.110078 (PMC3322079; doi:10.3201/eid1709.110078)
Supplement: Technical Appendix — Real-time PCR for Human Bocavirus. [file 11-0078-Techapp_2p.pdf]

Article DOI: http://dx.doi.org/10.3201/eid1709.110078

Bocavirus in Children with Respiratory Tract Infections

Technical Appendix

Real-time PCR for Human Bocavirus

To quantify the viral loads of human bocaviruses (HBoVs), a universal Taqman real-time PCR that can detect HBoV1–4 was performed. The primers/probe of the Taqman real-time PCR were designed to target the conserved regions of the viral protein (VP) 1/2 gene segment of HBoV1–4, amplifying a 113-bp fragment. The sequence of the forward primer was 5′-TGGMATTATTGGMTCMAGTTT-3′, corresponding to the HBoV1 st1 strain (GenBank accession no. DQ000495) nt 3316–3326, and that of the reverse primer was 5′-CACCTTTATTTGAGTTDGCA-3′, corresponding to the HBoV1 st1 strain nt 3309–3328. The sequence of the probe was 5′-HEX-AAGCGCGCCGTGGCTCCTGCTCT-BHQ-1–3′, corresponding to the HBoV1 st1 strain nt 3247–3269. Each 25-µL reaction mixture consisted of 2 µL of viral DNA, 0.5 µM each of the forward and reverse primers, and 0.3 µM of the probe labeled at its 5′ end with a reporter dye (HEX) and at its 3′ end with a nonfluorescent quencher. PCR was conducted for 10 cycles at 95°C for 15 s, 50°C for 30 s, and 72°C for 1 min, followed by 40 cycles of 10 s each at 95°C and 40 s each at 60°C. The real-time PCR was performed on a CFX 96 (Bio-Rad Laboratories Inc., Hercules, CA, USA). The baseline fluorescence threshold was manually adjusted on the basis of background fluorescence of the no template control reaction by using the Baseline Subtracted Curve Fit analysis mode in Bio-Rad CFX Manager Software. Positive was defined as an amplification curve exceeding baseline fluorescence with a corresponding cycle threshold value (not exceeding a 40-cycle run). A real-time fluorescence amplification curve lower than the baseline fluorescence was considered negative. Serial

dilutions of the pGEMT Easy Vector (Promega, Madison, WI, USA) containing the HBoV VP1/2 gene segment were used as quantification standards. The housekeeping gene GAPDH was detected as the internal control to monitor the quality of the specimens. The limit of detection of this assay was 10 copies/reaction.
